# Integrated Analysis Identifies a Nine-microRNA Signature Biomarker for Diagnosis and Prognosis in Colorectal Cancer

**DOI:** 10.3389/fgene.2020.00192

**Published:** 2020-03-20

**Authors:** Ziyang Di, Maojun Di, Weihua Fu, Qiang Tang, Yanwei Liu, Peijie Lei, Xinsheng Gu, Tong Liu, Min Sun

**Affiliations:** ^1^Department of General Surgery, Tianjin Medical University General Hospital, Tianjin Medical University, Tianjin, China; ^2^Department of General Surgery, Taihe Hospital, Hubei University of Medicine, Shiyan, China; ^3^The First Clinical School, Hubei University of Medicine, Shiyan, China; ^4^College of Basic Medical Sciences, Hubei University of Medicine, Shiyan, China; ^5^Hubei Key Laboratory of Embryonic Stem Cell Research, Taihe Hospital, Hubei University of Medicine, Shiyan, China; ^6^Department of Anesthesiology, Institute of Anesthesiology, Taihe Hospital, Hubei University of Medicine, Shiyan, China

**Keywords:** hsa-miRNA-200a, FOXA1, colorectal cancer, prediction model, prognosis, biomarker

## Abstract

**Background:**

Colorectal cancer (CRC) is the third most lethal and malignant type of cancer in the world. Abnormal expression of human microRNA-200a (hsa-miRNA-200a or miR-200a) has previously been characterized as a clinically noticeable biomarker in several cancers, but its role in CRC is still unclear.

**Methods:**

Three CRC miRNA expression datasets were integratively analyzed by Least Absolute Shrinkage and Selector Operation (LASSO) and Support Vector Machine-Recursive Feature Elimination (SVM-RFE) algorithms. Nine candidate miRNAs were identified and validated for diagnostic and prognostic capability with the prediction model. The potential roles of the tumor suppressor miR-200a-3p in invasion, migration, and epithelial-mesenchymal transition of CRC cells were elaborated by *in vitro* studies.

**Results:**

Nine miRNAs (miR-492, miR-200a, miR-338, miR-29c, miR-101, miR-148a, miR-92a, miR-424, and miR-210) were identified as potentially useful diagnostic biomarkers in the clinic. The overall accuracy rate of the nine miRNAs in the diagnostic model was 0.94, 0.89, and 0.978 in the testing, validation, and independent validation dataset, respectively. CRC patients in the GSE29622 cohort were separated by the prognostic model into the low-risk score group and the high-risk score group. The area under the receiver operating characteristic curve (AUC) was 0.872 and 0.783 for predicting the 1- to 10-year survival of CRC patients. The performance of the prognostic model was validated by an independent TCGA-Colon Adenocarcinoma (COAD) dataset with AUC values between 0.911 and 0.796 in predicting 1- to 10-year survival. Nomograms comprising risk scores, tumor stage, and TNM staging were generated for predicting 1-, 3-, and 5-year overall survival (OS) in the GSE29622 and TCGA-COAD datasets. Colony formation, invasion, and migration in DLD1 and SW480 cells were suppressed by overexpression of miR-200a-3p. Inhibition of miR-200a-3p function contributed to abnormal colony formation, migration, invasion, and epithelial–mesenchymal transition (EMT). miR-200a-3p binding sites were located within the 3′-untranslated region (3′-UTR) of the Forkhead box protein A1 (FOXA1) mRNA.

**Conclusion:**

We developed and validated a diagnostic and prognostic prediction model for CRC. miR-200a-3p was determined to be a potential diagnostic and prognostic biomarker for CRC.

## Introduction

Colorectal cancer is the third most commonly diagnosed cancer in both men and women worldwide (Siegel et al., 2017). The 5-year survival percentage for localized stage CRC reaches 90%. However, as CRC cells are transferred to the regional lymph nodes or different organs of the body, the 5-year survival rate declines from 71 to 13% in the United States (Siegel et al., 2016). The prognosis of CRC patients is dependent on the TNM stage of CRC and curative surgical intervention, which is applicable only for patients with CRC limited to primary tumor and regional lymph nodes. Moreover, the clinical performance, therapeutic efficacy, and prognosis of the patients are affected by many factors such as the epigenetic status and microenvironments that cause CRC heterogeneity (Linnekamp et al., 2015). It is widely accepted that diagnosis and resection at a precancerous or early stage based on effective biomarkers are the keys to the most patients being cured (Ribic et al., 2003; Kennedy et al., 2011). Furthermore, it is imperative to identify biomarkers that are essential for accurately monitoring patients’ progression and their response to treatment (Danielsen et al., 2018; Ozawa et al., 2018).

MicroRNAs (miRNAs) have been developed as novel biomarkers for early non-invasive or minimally invasive diagnosis of CRC (Gangadhar and Schilsky, 2010; Chen et al., 2015). Expression profiles of miRNAs in human colon cancer were utilized to determine the potential clinical relevance in several studies (Smits et al., 2011; Ma et al., 2012; Manceau et al., 2014; Wu et al., 2014; Kandimalla et al., 2018). A single model incorporating multiple biomarkers demonstrates improved performance and stability of prognostic value (Agesen et al., 2012; Marisa et al., 2013). However, further validation is still needed for the implementation of these miRNAs in standard CRC clinical practice, and the underlying mechanism remains to be elucidated (Huang et al., 2017; Lin et al., 2019).

The miR-200 family (miR-200a, -200b, -200c, -141, and -429) has been demonstrated as an inhibitor of epithelial-to-mesenchymal transition (EMT) and metastasis of cancer in colon (Hur et al., 2013). Chen et al. (2019) reported that miR-200a is a tumor suppressor in glioma by directly targeting forkhead box A1 (FOXA1). FOXA1 is a transcription factor that is essential for epithelial lineage differentiation, playing an oncogenic or tumor-suppressive role in human malignancies (Song et al., 2019). FOXA1 upregulates the expression of YAP, promoting CRC tumor growth (Ma et al., 2016). Our previous study confirmed that YAP mediates EMT (Sun et al., 2017a). *FOXA1* might be the potential target of miR-200a, regulating YAP-mediated EMT; however, the underlying mechanism in CRC remains unclear.

In this four-phase study, we developed a data processing system to solve the curse of dimensionality in high-dimensional gene expression data using LASSO and SVM. Different independent datasets were first integrated by using Fisher’s method to expand the sample size, and the integrated dataset was then screened for candidate miRNAs of CRC using a prediction model combining the LASSO and SVM models. The full-length 3′-UTR of human *FOXA1* mRNA was proven for the first time to be a direct target of miR-200a-3p in CRC. A multi-miRNA-based classifier with a logistic regression model was developed for CRC screening or early diagnosis and was validated with the Cox regression model for potential predictors of prognosis. In addition, the results of the present study demonstrate a potential data processing model for identifying novel biomarkers and candidate miRNA patterns in the detection and prognosis prediction of CRC.

## Materials and Methods

### Data Collection, Preprocessing, and Normalization

Public microarray datasets were extracted from the GEO and TCGA database. The checklist and pipeline for proper organization of the integrated analysis were determined following the reporting guidelines of microarray meta-analysis recommended by Ramasamy et al. (2008). Only original experimental studies to screen miRNAs that were differentially expressed (DE) between CRC and ANT in at least 40 human samples were included. Selection criteria, probe annotation, and data normalization were the same as described in previous reports (Sun et al., 2017a, b; Lin et al., 2019).

### Integrated Analysis of miRNA Expression Datasets

Differentially expressed miRNAs between CRC and ANT were determined by MetaOmics software^1^ in the MetaDE package (Wang et al., 2012). The filter thresholds of the mean and SD were set to 30% in integrated analysis. Fisher’s method was performed for statistically significant analysis to counterpoise the different stringencies of the methods. A permutation method with a modified *t-*test was used for the removal of the batch effect and estimation of the *P*-values (Tseng et al., 2012). *P-*value < 0.001 was used as the cutoff for statistically significant DE miRNAs.

### Selection of miRNAs for the Training Cohort

The candidate miRNAs were determined by a LASSO algorithm with penalty parameter tuning with 10-fold cross-validation and an SVM-Recursive Feature Elimination (RFE) algorithm (Qiu et al., 2017). In total, nine common candidate miRNAs were further selected for the training cohort.

### Development and Validation of the Logistic Prediction Diagnosis Model

The diagnosis model was developed for evaluation of the differential capacity of CRC and ANT in GSE49246 using logistic regression of the nine candidate miRNAs. Furthermore, candidate miRNAs were used to validate the results of the ROC curve analysis in an independent validation set (GSE115513) by forecasting the grades of CRC and ANT, as described previously (Sun et al., 2017b).

### Prognostic Index of the Nine-miRNA Signature and Validation on Prognosis of CRC Survival

A nine-miRNA prognostic signature model was developed by multivariate Cox hazard model analysis of GSE29622 data to estimate prognostic risk score (PRS) using the formula: PRS = Σ(C × EXP_miRNA_), where EXP was the FPKM value of the miRNA, and C was the regression coefficient for the corresponding miRNA in multivariate Cox hazard model analysis. The median PRS of the testing dataset GSE29622 was used to distinguish the high-risk cluster from the low-risk cluster. The prognostic performance of the miRNA signature model was evaluated by comparing the AUC of ROC curves. The association of miRNA signature with patient survival was analyzed, and the miRNA signature model was finally validated in an independent dataset, TCGA-COAD.

### Target Prediction of miRNA

The speculated targets of integrated-signature miRNAs, particularly for the tumor suppressor miRNA hsa-miR-200a, were predicted by four different target-prediction algorithms: TargetScan v7.1^2^, miRanda^3^, DIANA-TarBase v7.0^4^, and PicTar^5^ via the “miRNAtap” package (Dhawan et al., 2018).

### Cell Culture and Transfection of miR-200a-3p Mimic and Inhibitor

Human colon cancer cell lines DLD1 and SW480 and normal intestinal epithelium cell line NCM460 were provided by the Cell Bank of Wuhan University (Wuhan, China). hsa-miR-200a-3p mimic, inhibitor, mimic NC, and inhibitor-negative control were from RiboBio Co., Ltd. (Guangzhou, China). Cell culture and transient transfection were performed as described previously (Sun et al., 2017a; Lin et al., 2019).

### Tissue Collection

Colorectal cancer tissues and ANT (distance to cancer >5 cm) were sampled from sixty patients with primary CRC that was diagnosed by pathological assessment of tissues at Taihe Hospital of Hubei University of Medicine from January 2017 to December 2018. The study was authorized by the Research Ethics Committee of Hubei University of Medicine (Shiyan, Hubei, China). Informed consent was provided by all patients who participated in the present study.

### Total RNA Isolation and Quantitative Reverse Transcription Polymerase Chain Reaction (qRT-PCR)

Total RNA was isolated using Trizol reagent (Invitrogen, United States). The expression of mature miR-200a-3p was determined using qRT-PCR with the Bulge-Loop^TM^ miRNA qRT-PCR Primer Set and Control Primer Set (RiboBio, Guangzhou, China); 2 μg of RNA was used to synthesize cDNA using the RevertAid First Strand cDNA Synthesis Kit (Thermo, United States). qRT-PCR was performed using the FastStart Universal SYBR Green Master (Rox) (Roche, United States) in the ABI PRISM^®^ 7300 real-time PCR system (Applied Biosystems, Foster City, CA, United States). GADPH and U6 were added as endogenous controls, and melting curves were used to check non-specific amplifications. Relative expression level was calculated by the 2^–Δ^ ^ΔCt^ method. The primer sequences are shown in Supplementary Data Sheet S1.

### Western Blotting and Dual-Luciferase Reporter Assays, Colony Formation Assay, Wound Healing Assay *in vitro*, and Transwell Migration/Invasion Assay

Approximately 0.2 g CRC and paired normal tissue samples were ground in liquid nitrogen and lysed with RIPA lysis buffer (CelLytic, Sigma-Aldrich, United States) and proteinase inhibitor cocktail (Merck Millipore, United States). Total protein was extracted and immunoblotted as described previously (Sun et al., 2017a; Lin et al., 2019). The antibody used in the present study is shown in Supplementary Data Sheet S1. The dual-luciferase reporter assays, colony formation assay, wound healing assay *in vitro*, transwell migration/invasion assay, transfection reagents, primers, and western blot antibody are described in Supplementary Data Sheet S1.

### Statistical Analysis

All statistical analyses were performed using R/BioConductor (version 3.5.1) with two-tailed *P*-values. In the training phase, the dependent variable of the LASSO-logistic regression and SVM-RFE algorithm is dichotomized into two groups (tumor vs. normal), whereas the independent variable is the continuous expression values of 68 miRNAs.

In the testing phase, logistic regression is used for the diagnostic model of the binary variables, tumor and normal, and the independent variable is the continuous expression values of nine miRNAs. ROC was analyzed with R (“pROC,” “MASS,” “nnet,” “Daim,” and “epicalc” packages) to evaluate the diagnostic efficiency for the diagnostic model on the CRC outcome for the expression level of the nine candidate miRNAs. For Cox regression, the dependent variable is survival data showing the state of survival and the time of follow-up, whereas the independent variable is the continuous expression values of the nine miRNAs. Kaplan-Meier plots and ROC curve for the prognosis model were constructed using R (“survival,” “survminer,” “ggplot2,” and “survivalROC” packages). The median value was used as the cutoff value between low and high expression levels of each miRNA. The Cox proportional hazards regression model was applied to determine OS, and log-rank test was used to detect differences in OS.

For the validation phase, the nomogram prognostic model contains clinical features such as the total TNM Stage, T stage, N stage, M stage, and risk score. The clinical features (TNM Stage, T stage, N stage, M stage) are factor variables (categorical variables), and risk score is a continuous variable. The performance of the nomogram for the testing and validation data was evaluated by the concordance index and was distributed graphically with calibration plots using the “rms” package in R.

In the verification phase, ANOVA or Student’s *t*-test was used to detect significant differences between the groups with *P* < 0.05 as the threshold. The data from at least three independent experiments were averaged and presented as mean ± SD.

## Results

### Identification of Significantly Deregulated miRNAs

**FIGURE 1:**
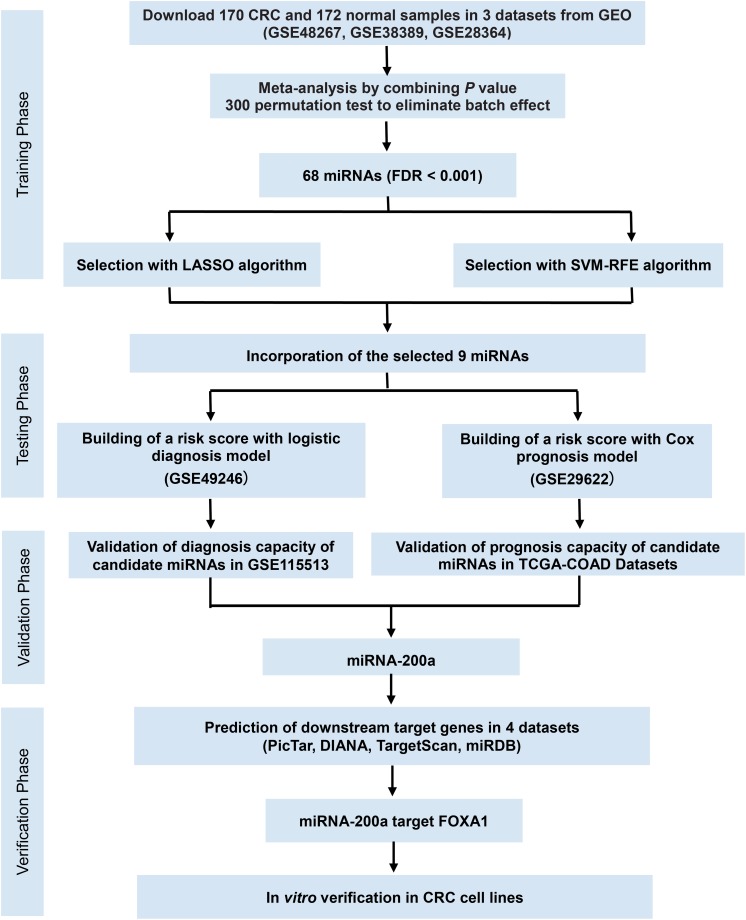
Study flowchart of the meta-analysis, bioinformatics analysis, and functional validation. *In silico* data analysis system includes the curation of three publicly available datasets, data preprocessing, meta-analysis, validation for diagnostic/prognostic values, and *in vitro* functional validation.

**FIGURE 2:**
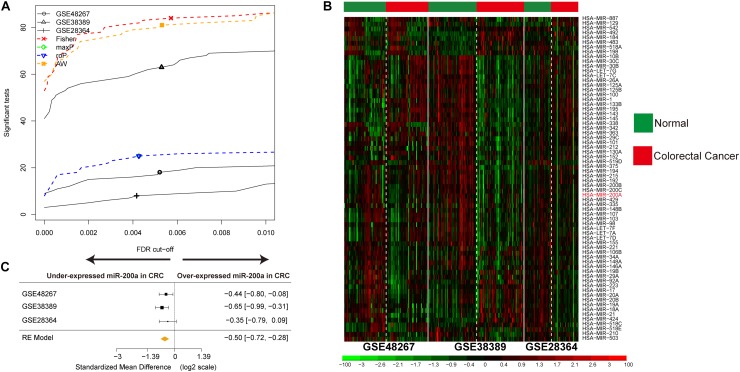
Identification of DE miRNAs in CRC and data integration. **(A)** The number of DE miRNAs represented as a function of false discovery rate (FDR) in the analysis of three different datasets and four different meta-analysis algorithms (maxP, Fisher roP, and adaptively weighted statistic). **(B)** Heat map and two-way hierarchical clustering based on 68 DE miRNAs between CRC and ANT samples in the training set. ANT (green) and CRC (red) samples fell into different groups. **(C)** Forest plot of hsa-miR-200a expression throughout all pooled training datasets.

Three CRC miRNA datasets consisting of 170 CRC samples and 172 ANT samples (Supplementary Table S1) were selected by integrated analysis (Figure 1), from which 135 miRNAs with steady DE patterns were identified by a moderated *t*-test with addition of a fudging parameter, and Fisher’s method with summarization of -log (*p*-value) across studies to run 300 selections (Wang et al., 2019). The effect size was merged, and a total of 68 DE miRNAs with profiles of DE miRNAs similar to those from Fisher’s method of combining *P* < 0.001 (Supplementary Table S2) were determined (Figure 2A). As expected, the CRC was differentiated from ANT samples by hierarchical clustering of the 68 DE miRNAs (Figure 2B).

### Screening of Candidate miRNAs

**FIGURE 3:**
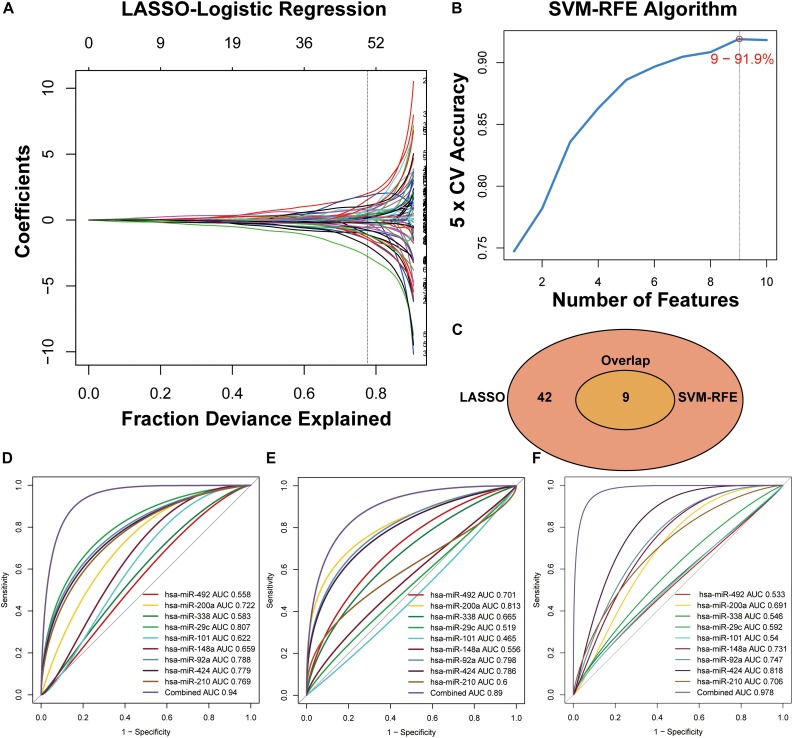
Two algorithms used for feature selection and ROC curve for the nine-miRNA signature for CRC diagnosis. **(A)** LASSO and **(B)** SVM-RFE algorithms in the training phase. **(C)** Venn diagram of incorporation of miRNAs that were selected from the LASSO or SVM-RFE algorithms in the training phase. **(D)** Development of a risk grade with logistic diagnosis model of GSE49246. **(E)** Validation of the diagnostic capacity of candidate miRNAs in GSE115513. **(F)** Validation of the diagnostic capacity of candidate miRNAs in the independent validation cohort.

The miRNAs that were selected by the LASSO (Figure 3A) and SVM-RFE (Figure 3B) algorithms were merged, among which nine miRNAs that were overlapped in both algorithms (Figure 3C, Supplementary Table S3 and Supplementary Figure S1) and constituted a minimum candidate list for model prediction of CRC samples (Figures 3A–C).

### Diagnostic Capacity of Candidate miRNAs for CRC in GEO Datasets

A total of 80 testing-phase samples in GSE49246 and 1513 validation-phase samples in GSE115513 were differentiated into the CRC and normal groups using a linear logistic regression model of nine miRNAs. A DRS was estimated and weighted with the coefficients by the univariate linear regression model to evaluate the performance of nine miRNAs for prediction of CRC in the GSE49246 cohort. The DRS was estimated by the formula: DRS = (−5.1928 × EXP_hsa–miR–__492_) + (0.1885 × EXP_hsa–miR– 200__a_) + (−3.5434 × EXP_hsa–miR–__338_) + (−9.1146 × EXP_hsa–miR– 29__c_) + (0.8177 × EXP_hsa–miR–__101_) + (−1.3238 × EXP_hsa–miR– 148__a_) + (2.9460 × EXP_hsa–miR–__92__a_) + (−1.2699 × EXP_hsa–miR– 424_) + (2.7264 × EXP_hsa–miR–__210_). ROC curve analysis indicated high categorization accuracy of a panel of nine miRNA signatures in the testing set GSE49246 (AUC = 0.94) (Figure 3D), in the validation set GSE115513 (AUC = 0.89) (Figure 3E), and in our independent validation set (AUC = 0.978) (Figure 3F and Supplementary Table S4).

### Prognostic Capacity of Candidate miRNAs for CRC in GEO and TCGA Datasets

Significant associations of candidate miRNAs with the survival of CRC patients were analyzed by multivariate Cox regression in 80 testing-phase GSE29622 samples (Supplementary Table S4). PRS was estimated using the nine-miRNA signature model as follows: PRS = (0.1170 × EXP_hsa–miR–__492_) + (0.3496 × EXP_hsa–miR–__92__a_) + (0.3640 × EXP_hsa–miR–__424_) + (−0.5316 × EXP_hsa–miR–__29__c_) + (0.0573 × EXP_hsa–miR–__101_) + (0.0882 × EXP_hsa–miR–__338_) + (−0.5038 × EXP_hsa–miR–__200__a_) + (0.1920 × EXP_hsa–miR–__210_) + (0.2397 × EXP_hsa–miR–__148__a_), where EXP was the FPKM value of the miRNA.

**FIGURE 4:**
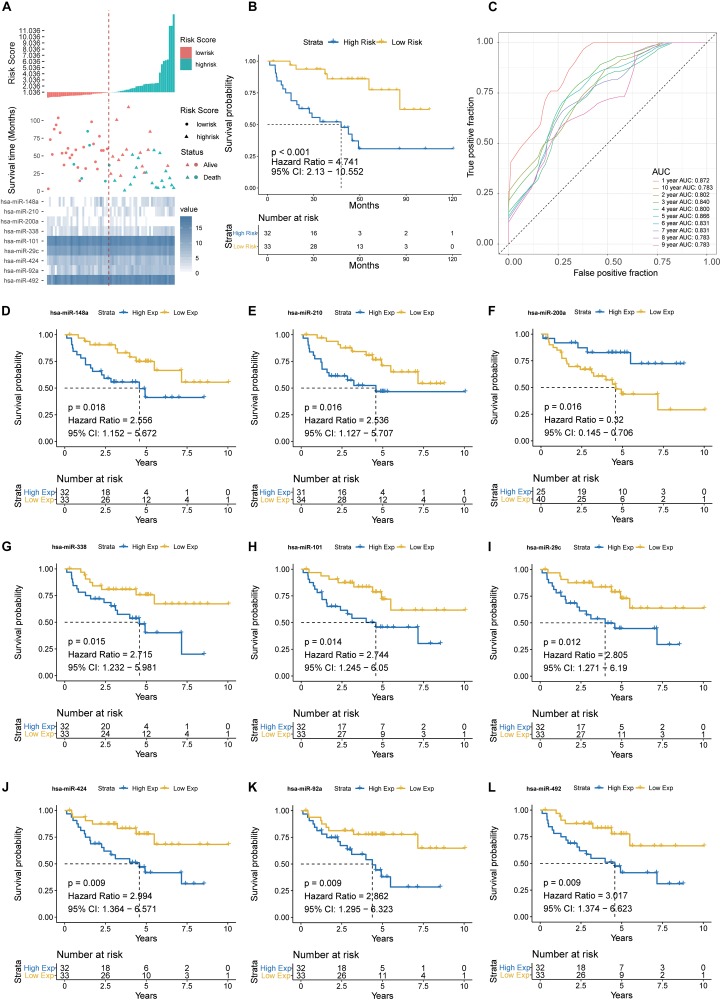
Detection performance of the nine-miRNA signature in GSE29622 data. **(A)** Risk grade analysis of the nine-miRNA signature of CRC. Risk grade of miRNA signature (Top); duration of cases (Middle); low- and high-risk clusters for the nine miRNAs (Bottom). **(B)** Survival analysis of the high-risk cluster and the low-risk cluster with Kaplan–Meier curves. **(C)** Detection effectiveness of the nine-miRNA signature for survival time. ROC curves of the nine-miRNA signature were used to predict 1- to 10-year survival. Independent detection power (efficiency) of individual miRNAs in the nine-miRNA signature. **(D)** hsa-miR-148a, **(E)** hsa-miR-210, **(F)** hsa-miR-200a, **(G)** hsa-miR-338c, **(H)** hsa-miR-101, **(I)** hsa-miR-29c, **(J)** hsa-miR-424, **(K)** hsa-miR-92a, and **(L)** hsa-miR-492. The *x*-axis represents overall survival time, whereas the *y*-axis indicates OS probabilities.

The prognostic performance of the nine-miRNA signature model was characterized using the GSE29622 dataset. The median PRS (1.036) was used to classify CRC patients into a low-risk group (*n* = 33) and a high-risk group (*n* = 32) (Figure 4A). As shown in Figure 4B, the high-risk group had significantly shorter survival time or lower survival probability compared with the low-risk group (HR, 4.741, 95% CI, 2.13–10.55, log-rank test *p* = 0.0002). The AUC for predicting the 1- to 10- year survival of CRC patients was between 0.783 and 0.872 (Figure 4C). High expression of hsa-miR-200a and low expression of hsa-miR-492, hsa-miR-92a, hsa-miR-424, hsa-miR-29c, hsa-miR-101, hsa-miR-338, hsa-miR-210, and hsa-miR-148a were associated with longer predicted survival (Figures 4D–L). The results suggested the nine-miRNA signature enabled effective prediction of the prognosis of CRC patients.

### Validation of Prognostic Performance of the Nine-miRNA Signature in TCGA-COAD

**FIGURE 5:**
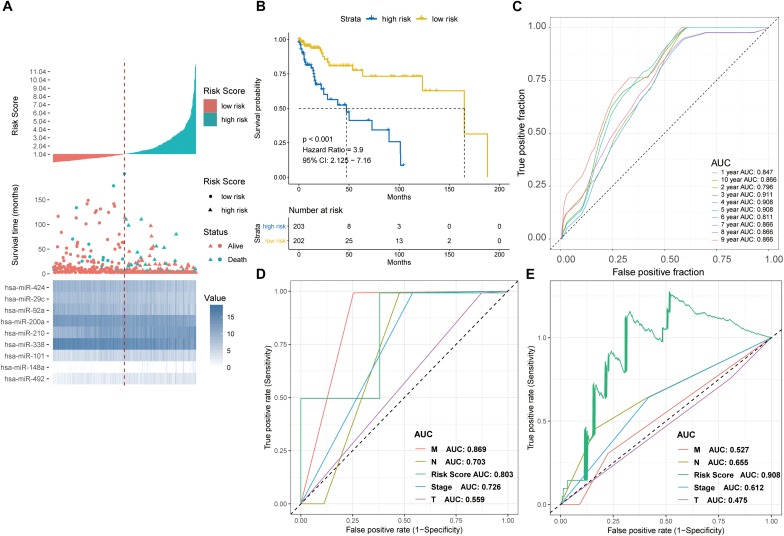
Detection performance of the nine-miRNA signature in the TCGA-COAD test cohort and ROC analysis for comparison of performance of risk grade with the clinicopathological factors. **(A)** Risk grade analysis of the nine-miRNA signature in COAD data. Risk grade of miRNA signature (Top); duration of cases (Middle); low- and high-risk clusters for the nine miRNAs (Bottom). **(B)** Survival analysis of the high- and the low-risk clusters by Kaplan–Meier curves. **(C)** Detection effectiveness of the nine-miRNA signature for survival time. ROC curves of the nine-miRNA signature were applied to predict 1- to 10-year survival. ROC analysis for comparison of performance of risk grade with the clinicopathological factors in **(D)** GSE29622 and **(E)** TCGA-COAD data.

The prognostic performance of the nine-miRNA prediction model for CRC was validated using the validation dataset TCGA-COAD. The CRC patients were classified into the high-risk group (*n* = 203) and the low-risk group (*n* = 203) using PRS and the median PRS as cut-off criteria (Figure 5A). The high-risk group had significantly shorter survival time or lower survival probability compared with the low-risk group (HR, 3.9, 95% CI, 2.125-7.16, log-rank test *p* < 0.001) (Figure 5B). The AUC for predicting 1- to 10- year survival of CRC was between 0.796 and 0.911 (Figure 5C). The results were consistent with those of the testing set (Figures 4, 5), supporting the effectiveness of the nine-miRNA signature in predicting the prognosis of CRC.

The effect of each clinicopathological feature on survival was analyzed by Cox regression to evaluate whether the prognostic classifier acted as an independent indicator in CRC patients. As shown in Table 1, after multivariable adjustments for clinicopathological factors, the nine-miRNA-based classifier was demonstrated to be a powerful and independent factor in the testing cohort of 65 cases (HR 1.232, 95% CI 1.115–1.363, *P* < 0.001) and in the validation set of 406 cases (HR 1.455, 95% CI 1.287–1.645; *P* < 0.001).

**TABLE 1:** Univariate and multivariate analyses of clinicopathological factors and nine-miRNA classifier with OS in GSE29622 and TCGA COAD cohorts.

	Univariate analysis^a^		Multivariate analysis^b^	
	HR (95% Cl)	*P-*value	HR (95% CI)	*P-*value
**GSE29622 testing set (*n* = 65)**				
Gender (male vs. female)	1.586 (0.682–3.687)	0.284		
Pathologic T stage (T3–4 vs. T1–2)	1.678 (0.394–7.141)	0.484	0.559 (0.099–3.162)	0.511
Pathologic N stage (N0 vs. N1 vs. N2)	**2.744 (1.562–4.820)**	**<0.001**	1.322 (0.559–3.128)	0.525
Pathologic M stage (present vs. absent)	**5.059 (2.243–11.410)**	**<0.001**	**3.09 (1.231–7.757)**	**0.016**
TNM stage (III and IV vs. I and II)	**5.290 (1.813–15.436)**	**0.002**	2.331 (0.421–12.911)	0.332
Risk Score (continuous value)	**1.272 (1.159–1.396)**	**<0.001**	**1.232 (1.115–1.363)**	**<0.001**
**TCGA-COAD validation set (*n* = 406)**				
Gender (male vs. female)	1.299 (0.727–2.321)	0.376		
Pathologic T stage (T3–4 vs. T1–2)	**3.081 (1.393–6.818)**	**0.005**	2.188 (0.919–5.210)	0.077
Pathologic N stage (N0 vs. N1 vs. N2)	**1.541 (1.092–2.175)**	**0.014**	1.654 (0.897–3.050)	0.107
Pathologic M stage (present vs. absent)	**3.919 (2.080–7.383)**	**<0.001**	**3.335 (1.511–7.358)**	**0.003**
TNM stage (III and IV vs. I and II)	**1.839 (1.027–3.292)**	**0.040**	0.602 (0.189–1.916)	0.390
Risk score (continuous value)	**1.415 (1.262–1.587)**	**<0.001**	**1.455 (1.287–1.645)**	**<0.001**

*^a^The data were subjected to the Cox proportional hazards regression model. ^b^Multivariate analysis used stepwise addition and removal of clinical covariates found to be associated with survival in univariate models (P < 0.05), and final models include only those covariates that were significantly associated with survival (Wald statistic, P < 0.05). Bold indicate statistically significant values (P < 0.05).*

Time-dependent ROC curve analysis revealed that the nine-miRNA signature was a better predictor of survival compared with clinicopathologcial factors in both the testing and validation sets (Figures 5D,E).

## Building a Predictive Nomogram and Calibration Plot

A predictive model applicable for clinics was built using a nomogram to predict an individual’s 1-, 3-, and 5-year survival, taking into consideration clinicopathological covariates. According to multivariate analysis of OS (Table 1), a nomogram with predictors including PRC, TMN stage, and pathological stage was used to predict the 1-, 3-, 5-, and 7- year survival in both the testing cohort (Supplementary Figure S2A) and validation cohort (Supplementary Figure S2B). The concordance index was 0.827 (0.761–0.893) for the testing cohort (Supplementary Figure S2C) and 0.837 (0.778–0.895) for the validation cohort (Supplementary Figure S2D), suggesting that the calibration plots effectively predicted the 1-, 3-, and 5-year OS rate.

### Association of Downregulated miR-200a-3p With Upregulated FOXA1 in CRC

**FIGURE 6:**
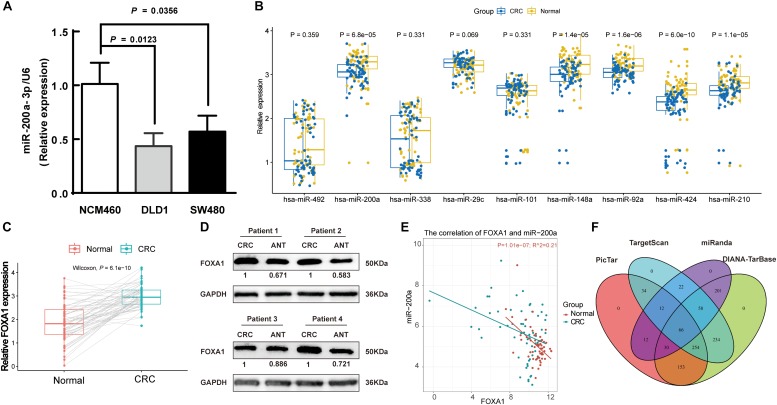
Negative association of expressions of miR-200a-3p with *FOXA1* in primary CRC tumors. **(A)** Expression of miR-200a-3p measured in triplicate in two colon cancer cell lines, DLD1 and SW480, and the normal NCM460 cells. **(B)** Significantly declined miR-200a-3p, miR-148a, miR-92a, miR-424, and miR-210 in primary human CRC tissues in comparison with ANT. **(C)** Significantly increased FOXA1 in primary CRC samples in comparison with ANT. Significance was statistically analyzed by Student’s *t*-test. **(D)** The levels of FOXA1 protein in four pairs of CRC tissues. **(E)** Scatter plots indicating the negative association between miR-200a-3p and FOXA1 mRNA levels. **(F)** Venn diagrams of putative miR-200a targets predicted by PicTar, TargetScan (v7.1), miRanda, and DIANA-TarBase (v7.0).

A decreasing mode in the miR-200a levels was observed in human CRC (Figure 2C). The levels of miR-200a-3p in DLD1 and SW480 cell lines were lower than those in the normal intestinal epithelium cell line NCM460 (Figure 6A). qRT-PCR (normalized with U6) was used to measure mature miR-200a-3p level in the independent validation sample cohort (*n* = 60). The results indicated that the expression of miR-200a-3p was significantly (*P* < 0.05) decreased in the ANT-related tumors (Figure 6B). Both qRT-PCR and Western blot analyses demonstrated significant upregulation of *FOXA1* in CRC tissues compared with ANT (Figures 6C,D). The miR-200a-3p expression levels were significantly and inversely associated with FOXA1 levels in both CRC tissues and normal samples (*R*^2^ = 0.21, *P* = 1.01E-07) (Figure 6E) in our independent validation cohort, but they were not associated with FOXA1 levels in the TCGA colon or rectal cancer cohort (Supplementary Figure S3).

A total of 66 common targets of miR-200a were identified from both prediction algorithms and experimentally supported databases using four publicly available algorithms with high-stringency (Figure 6F and Supplementary Table S5).

### Inhibition of miR-200a in Clone Formation, Migration, and Invasion of Colon Cancer Cells

**FIGURE 7:**
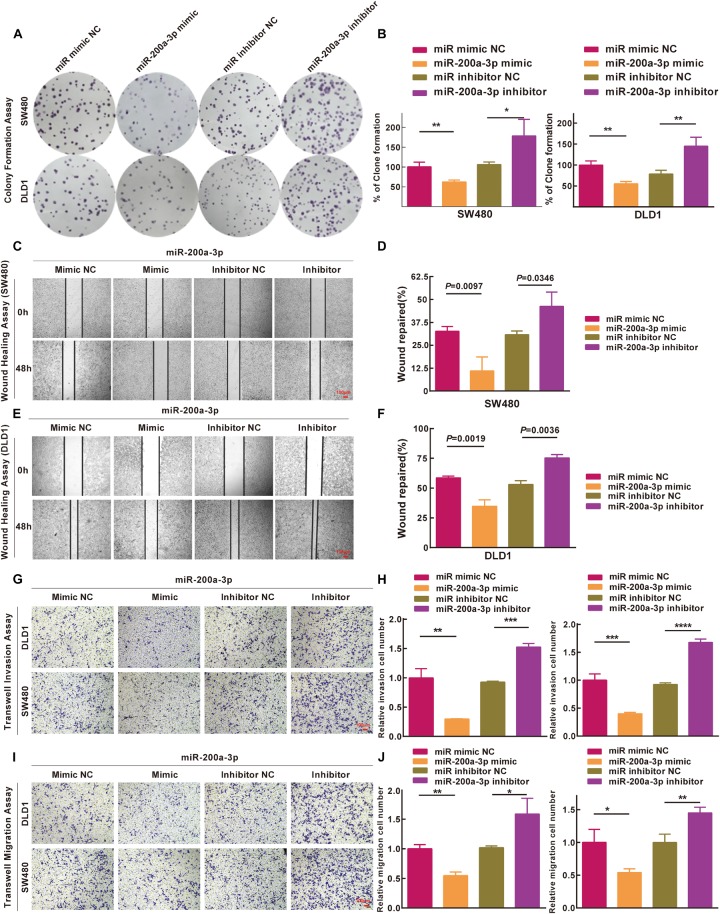
Inhibitions of miR-200a-3p to formation, movement, and penetration in SW480 and DLD1 cell clone *in vitro*. **(A,B)** Typical photomicrographs and quantifications of clone formation assay in SW480 and DLD1 cells at 48 h after transfection. **(C–F)** Photomicrographs of wound healing assay in SW480 and DLD1 cells at 48 h after transfection. **(G)** Transwell invasion assays of DLD1 and SW480 cells. Bar = 100 μm. **(H)** Total number of cells in five fields was estimated manually. **(I)** Transwell migration assays of DLD1 and SW480 cells with different miRNAs. Bar = 100 μm. **(J)** Total number of cells in five fields was determined manually. Mean ± SD was used, and statistical significance analysis was performed with one-way ANOVA. ^∗^*P* < 0.05. ^∗∗^*P* < 0.01. ^∗∗∗^*P* < 0.001.

Based on the results of the integrated analysis and *in vitro* experiments, we hypothesized that the reduced expression of miR-200a-3p might accelerate CRC tumorigenesis via targeting *FOXA1*. We transfected SW480 and DLD1 cells with miR-200a-3p mimic, miR mimic NC, miR-200a-3p inhibitor, miR inhibitor NC to determine the role of miR-200a-3p in clone formation. Clonogenic assay revealed that the clonogenic survivals of SW480 and DLD1 cells were reduced by miR-200a-3p compared with the NC groups, whereas phenotype was reversed by miR-200a-3p inhibitor (Figures 7A,B). The wound healing assay showed that miR-200a-3p overexpression suppressed and miR-200a-3p inhibitor enhanced migration of CRC cells (Figures 7C–F). The transwell assay showed that miR-200a-3p overexpression suppressed and miR-200a-3p inhibitor enhanced invasion of CRC cells (Figures 7G–J). These data indicated that miR-200a suppressed the proliferation, migration, and invasion of CRC cells.

### miR-200a-3p Modulates FOXA1 and YAP1 Expression via Targeting Human FOXA1 3′UTR and Inhibits EMT of Colon Cancer Cells

**FIGURE 8:**
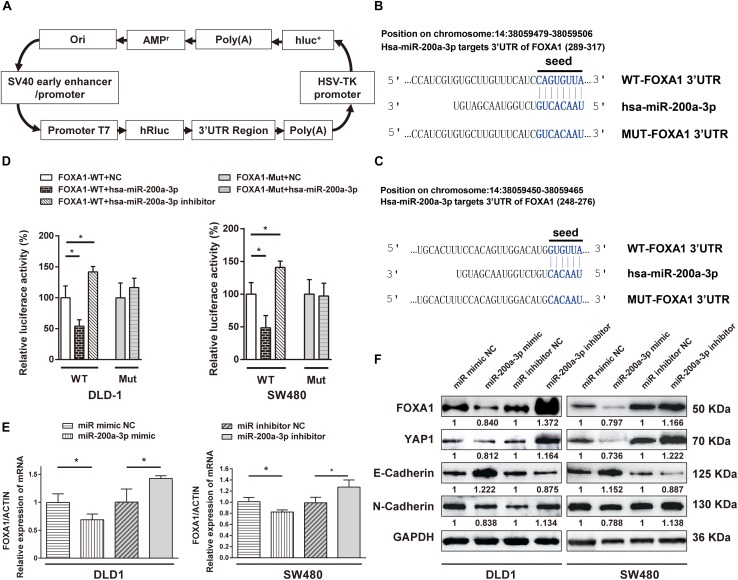
The 3′-UTR of FOXA1 mRNA is targeted by miR-200a-3p. **(A)** Schematic diagram of dual-luciferase reporter vector. **(B,C)** The 3′-UTR of FOXA1 mRNA, harboring two miR-200a-3p cognate sites. **(D)** Relative luciferase activity of reporter plasmids with wild-type or mutant FOXA1 3′-UTR in DLD1 and SW480 cells co-transfected with negative control (NC) or miR-200a-3p mimic or miR-200a-3p inhibitor. **(E)** Expression of FOXA1 mRNA in DLD1 and SW480 cells transfected with the indicated miRNAs. **(F)** Western blotting of FOXA1, YAP, E-cadherin, and N-cadherin in DLD1 and SW480 cells transfected with different miRNAs. Mean ± SD from triplicates is used, and statistical significance was determined by Student’s *t*-test. ^∗^*P* < 0.05.

*FOXA1* 3′-UTR was cloned into a luciferase reporter plasmid (Figure 8A), and expression of the adjacent hRluc coding region was quantified to evaluate miR-200a-3p-regulated *FOXA1* expression. Searching different databases revealed that *FOXA1* is a predicted target of miR-200a-3p that presents oncogenic properties. *FOXA1* harbors two conserved miR-200a-3p cognate sites: nucleotides 310-317 and 271–276 of *FOXA1* 3′-UTR (Figures 8B,C). The luciferase reporter plasmid FOXA1-3′-UTR or mutant reporter plasmids with point mutations in the putative miR-200a-3p binding sites were co-transfected with miR-200a-3p mimics or miR mimic NC and inhibitors, separately. The results showed that the luciferase activity of reporter plasmid with wild type *FOXA1* 3′-UTR was significantly inhibited by miR-200a-3p but increased by miR-200a-3p inhibitor (Figure 8D, *P* < 0.05). However, no significant change was observed in reporter plasmid containing mutated *FOXA1* 3′-UTR (i.e., MUT-FOXA1-3′-UTR). These results suggested that miR-200a-3p negatively regulated *FOXA1* expression by directly binding to the predicted binding site(s) in the *FOXA1* 3′-UTR.

DLD1 and SW480 cells were transfected with miR-200a-3p mimic, miR mimic NC, miR-200a-3p inhibitor, and inhibitor NC to verify the direct regulation of *FOXA1* expression by miR-200a-3p. Both qRT-PCR and Western blotting revealed that the expression level of *FOXA1* was suppressed in miR-200a-3p-overexpressed cells but regained in miR-200a-3p inhibitor-treated cells (Figures 8E,F). Therefore, miR-200a-3p negatively regulated FOXA1 levels in CRC cells. In addition, the results showed that miR-200a-3p negatively regulated expression of N-cadherin and YAP1 proteins and positively regulated E-cadherin expression (Figure 8F), suggesting that miR-200a-3p inhibited EMT of CRC cells.

## Discussion

Integrated analysis that unites cross-platform normalization of gene profiling and neglects the unavailability of raw data has been widely applied to detect DEGs at the mRNA and miRNA levels in CRC (Sun et al., 2017a, b; Lin et al., 2019). In the present study, the microarray data were analyzed by meta-analysis to detect candidate miRNAs that were potential diagnostic and prognostic markers. Further, a novel diagnostic and prognostic model based on candidate miRNAs was developed and validated for OS prediction of CRC in both testing and validation cohorts.

The novel tool successfully differentiated patients into high-risk and low-risk clusters with significant differences in OS. The classifier was proven to predict the survival of patients with CRC significantly better than other clinicopathological risk factors. When stratified by these, the nine-miRNA-based classifier appears to be an effective prognostic model to provide value supplementary to clinicopathological characteristics. The nine-miRNA signature was also demonstrated to be independent of other clinicopathological factors. In addition, the prognostic scores can be easily calculated according to the prognostic-score equation, and the levels of the nine miRNAs in the signature can be easily determined using quantitative real-time PCR or other customized test methods. Risks and prognosis can be effectively predicted by the prognostic scores. Therefore, the prognostic score model is easily applied by oncologists in clinical practice.

A comprehensive and practical nomogram was developed to predict individual survival rates, providing opportunities for clinicians to rank patients according to these valid tools. miR-200a-3p is the only tumor-suppressor miRNA of the nine-miRNA-based classifier and inhibits expression of *FOXA1*, *YAP1*, and N-Cadherin but enhances E-Cadherin expression in colon cancer cells. The expression of *FOXA1* was directly regulated by miR-200a-3p binding to the 3′-UTR of *FOXA1*.

Zhang et al. (2013) used a tool-integrated LASSO Cox regression model with multiple miRNAs to develop a molecular classifier based on a core set of six miRNAs for predicting the 5-year disease-free survival for patients with stage II CRC. However, previous studies on miRNA diagnostic and prognostic prediction were restricted by the small number of miRNAs screened, small sample sizes, lack of independent validation, and utilization of improper statistical methods for miRNA microarray data mining. In the present study, all nine candidate miRNAs were detected by SVM-RFE instead of LASSO; this difference might be caused by the inherent characteristics of each set of algorithms. It should be noted, however, that the nine miRNAs selected by integrated functions of LASSO and SVM-RFE were further validated, suggesting the feasibility of the integration strategy. Our model still needs to be validated with real samples in different oncology centers. In addition, all nine miRNAs except miR-101 were newly detected to be associated with CRC. Further characterization of molecules such as miR-210 will provide novel insights into CRC occurrence and advancement, leading to the identification of potential therapeutic targets for patients with CRC.

A number of miRNAs have been detected to predict the survival of patients with CRC (Balaguer et al., 2011; Zhang et al., 2013; Hur et al., 2015; Vychytilova-Faltejskova et al., 2016; Pettit et al., 2017; Toiyama et al., 2017; Ji et al., 2018; Kandimalla et al., 2018). Particularly, miR-492, miR-200a, miR-338, miR-29c, miR-101, miR-148a, and miR-92a have proven to be correlated with the prognosis or therapeutic outcome of patients with CRC (Gaedcke et al., 2012; Kuo et al., 2012; Schee et al., 2012; Tsai et al., 2013; Yong et al., 2013; Kim et al., 2014; Pichler et al., 2014; Sun et al., 2014; Xue et al., 2014; Chen et al., 2018; Fu et al., 2018; Igder et al., 2019). miR-492 is a rectal cancer-specific miRNA that was the single most upregulated (up to 16-fold higher) in rectal cancer and was never detected for colon cancers (Gaedcke et al., 2012). In an effort to characterize miRNAs related to aggressiveness and E-cadherin expression, Pichler et al. (2014) revealed low expression level of miR-200a as an independent prognostic factor pertaining to cancer-specific survival, contrary to the expression of EMT-related genes in cancer stem cell-enriched spheroid growth conditions. Yong et al. (2013) proposed that a three-miRNA (miR-193a, miR-23a, and miR-338) classifier appeared to be a potential blood biomarker for early detection of CRC. Xue et al. (2014) reported that miR-338-3p inhibited colorectal carcinoma cell invasion and migration by attacking smoothened. Kuo et al. (2012) reported significant downregulation of miR-29a and miR-29c in early-recurring CRC patients and proposed miR-29a/c as promising biomarkers for CRC early recurrence. Yang et al. (2019) demonstrated that miR-101 inhibited colon cancer by targeting CREB1. Tsai et al. (2013) discovered that hsa-miR-148a inhibited tumorigenesis by suppressing colon cancer cell multiplication and migration but not invasion, resulting in an accumulation of the G2 population. Therefore, hsa-miR-148a might be a simple and accountable biomarker for the detection of postoperative early relapse in patients with CRC after radical resection (Tsai et al., 2013). Overexpression of miR-92a in HCT116 cells was demonstrated to facilitate migration, proliferation, and resistance to apoptosis and to modify F-actin organization, leading to the enhanced oncogenicity (Alcantara and Garcia, 2019).

Chen et al. (2012) demonstrated that miR-424 overexpression significantly abrogated invasive potential, acting as a direct tumor suppressor. In the present study, we found that only a high expression level of miR-200a-3p in tumors was significantly associated with the enhanced OS of CRC patients, indicating a negative correlation of miR-200a-3p to CRC progression. Pichler et al. (2014) found inverse expression of EMT-related genes caused by abnormal expression of miR-200a, but the study did not explore its potential mechanism in CRC.

Considering the convenience of clinical application, miR-200a-3p was selected for function validation in our study. We confirmed downregulation of miR-200a in CRC tissues, which was substantially linked to poor survival of CRC patients. miRNA target analysis and luciferase reporter assays suggested that the miR-200a-3p-mediated regulatory mechanism in colon cancer cells was inhibition of *FOXA1* expression by targeting the 3′-UTR of *FOXA1* mRNA. Over-expression of miR-200a-3p significantly suppressed cellular proliferation. Furthermore, ectopic overexpression of miR-200a-3p reduced cell migration and invasion and enhanced expression of E-cadherin, suggesting the potential role of miR-200a-3p in CRC metastasis. To the best of our knowledge, this is the first meta-analysis to reveal the comprehensive mechanism underlying malignant development of CRC caused by loss of miR-200a-3p, which unleashes expression of *FOXA1*. FOXA1 is a transcription factor. *FOXA1* knockdown distinctly suppressed cell proliferation and promoted apoptosis in SW480 and HCT116 CRC cells. The expression of YAP in SW480 and HCT116 cells was also prominently downregulated by *FOXA1* knockdown to reduce the tumor growth of CRC (Ma et al., 2016), consistent with our previous study that confirmed inhibition of down-expression of YAP1 to the EMT in a manner associated with CRC invasion *in vitro* (Sun et al., 2017a). The Hippo/YAP signature was greatly enriched for FOXA1 binding sites, ranking second among all transcription factors analyzed by Fitamant et al. (2015). Therefore, the interaction between FOXA1 and YAP1 represents the balance between tumor maintenance and cell discrimination (Fitamant et al., 2015). Further study is needed to reveal more functions of miR-200a-3p in CRC carcinogenesis and development by targeting the *FOXA1/YAP1* signal pathway.

In the current study, we found that expression of miR-200a-3p was significantly decreased in ANT-related tumors and a significant upregulation of *FOXA1* in CRC tissues compared with ANT. The miR-200a-3p expression levels were significantly and inversely associated with FOXA1 levels in both CRC tissues and normal samples in our independent validation cohort, but they were not associated with FOXA1 levels in the TCGA colon or rectal cancer cohort. Different ethnic groups, genetic testing method, dietary habits, and culture may be responsible for the inconsistencies between the TCGA datasets and our independent validation cohort. Firstly, in terms of ethnic group, the original data source of the independent validation cohort was Asian, whereas the data source for TCGA datasets is Caucasian. Second, the data are detected in different ways. The assay used in the independent validation cohort is qRT-PCR, and the unit is the ratio of the internal parameters. The data of the TCGA datasets is sequencing data, and the unit of measurement is RPKM (Reads Per Kilobase of exon Model per Million mapped reads). Third, CRCs from patients embedded in geographically diverse populations and cultures reflect substantially different dietary exposures, extended over the whole life course. These differences may account for the inconsistency between the expression of miR-200a and FOXA1 in TCGA and that of the independent validation cohort. It also should be pointed out that there was no significant difference in the FOXA1 expression in four patients at different stages in our validation cohort. These issues and the underlying molecular mechanism deserve further study in the future.

In conclusion, the integrated analysis of miRNome profiling of human normal tissues and CRC tissues was performed to explore the roles of miRNAs in CRC in this study. In total, nine miRNAs (hsa-miR-492, hsa-miR-200a, hsa-miR-338, hsa-miR-29c, hsa-miR-101, hsa-miR-148a, hsa-miR-92a, hsa-miR-424, and hsa-miR-210) were proven to be promising diagnostic and prognostic markers in the clinic. Upregulation of miR-200a-3p suppressed the proliferation, migration, invasion, and EMT of colon cancer cells by targeting the *FOXA1/YAP1* signal pathway. Targeting miR-200a-3p might serve as a novel therapeutic approach for the treatment of CRC patients.

## Data Availability Statement

Publicly available datasets were analyzed in this study. This data can be found at The Cancer Genome Atlas and Gene Expression Omnibus: GSE48267 (http://www.ncbi.nlm.nih.gov/geo/query/acc.cgi?acc=GSE48267); GSE38389 (http://www.ncbi.nlm.nih.gov/geo/query/acc.cgi?acc=GSE38389); GSE- 28364 (http://www.ncbi.nlm.nih.gov/geo/query/acc.cgi?acc=GSE28364); GSE49246 (http://www.ncbi.nlm.nih.gov/geo/query/acc.cgi?acc=GSE49246); GSE115513 (http://www.ncbi.nlm.nih.gov/geo/query/acc.cgi?acc=GSE115513); GSE29622 (http://www.ncbi.nlm.nih.gov/geo/query/acc.cgi?acc=GSE29622); and TC GA-COAD (https://portal.gdc.cancer.gov/).

## Ethics Statement

The studies involving human participants were reviewed and approved by the Research Ethics Committee of Hubei University of Medicine (Shiyan, Hubei, China). The patients/participants provided their written informed consent to participate in this study.

## Author Contributions

ZD, MD, WF, and QT participated in the research design. YL, PL, TL, and MS performed the data analysis. TL, XG, and MS wrote or contributed to the writing of the manuscript. All authors read and approved the final version of the manuscript.

## Conflict of Interest

The authors declare that the research was conducted in the absence of any commercial or financial relationships that could be construed as a potential conflict of interest.

## Footnotes

1 http://www.pitt.edu/~tsengweb/MetaOmicsHome.htm

2 http://www.targetscan.org/vert_71/

3 http://www.microrna.org/microrna/home.do

4 http://diana.imis.athena-innovation.gr/DianaTools/index.php?r=tarbase/index/

5 http://pictar.mdc-berlin.de/cgi-bin/PicTar_vertebrate.cgi

## Supplementary Material

FIGURE S1 Cross-validation for tuning parameter selection in the LASSO logistical model **(A)** and SVM-RFE model **(B)**.

FIGURE S2 Establishment and validation of the composite clinicopathologic-miRNA nomogram for OS prediction. Nomograms for OS prediction in **(A)** testing cohort GSE29622 and **(B)** validation cohort TCGA-COAD. Calibration curve for the OS nomogram model in **(C)** testing cohort GSE29622, and **(D)** validation cohort TCGA-COAD. The dashed line represents the ideal nomogram, and the solid line represents the observed nomogram.

FIGURE S3 Scatter plots indicating the association between miR-200a and FOXA1 mRNA expression levels in **(A)** TCGA-COAD and **(B)** TCGA-Rectal cancer.

TABLE S1 Characteristics of the public microarray datasets used in this study.

TABLE S2 MiRNA-level results in integrated analysis of three datasets.

TABLE S3 Differentially expressed miRNAs between CRC and ANT selected using the LASSO and SVM-RFE algorithms.

TABLE S4 Clinical features of the CRC patients in the testing set (GSE29622), validation set (TCGA-COAD), and independent validation set.

TABLE S5 Sixty-six consensus target genes summarized by four different target prediction algorithms.

DATA SHEET S1 Supplementary experimental procedures.

## Abbreviations

AUC: area under the receiver operating characteristic curve
COAD: colon Adenocarcinoma
CRC: colorectal cancer
DRS: diagnosis risk score
EMT: epithelial mesenchymal transition
FOXA1: forkhead box protein A1
FPKM: fragments per kilobase of transcript per Million mapped reads
GEO: gene expression omnibus
hsa-miRNA-200a or miR-200a: human microRNA-200a
LASSO: least absolute shrinkage and selector operation
NC: negative control
OS: overall survival
ANT: paired adjacent normal tissue
RIPA: radioimmunoprecipitation assay
ROC: receiver operating characteristic
SD: standard deviation
SVM: support vector machine
SVM-RFE: support vector machine-recursive feature elimination
TCGA: the cancer genome atlas
YAP: YES-associated protein.
