# Supplementary figures and images for: Integrated Analysis Identifies a Nine-microRNA Signature Biomarker for Diagnosis and Prognosis in Colorectal Cancer
Source: Front Genet. 2020 Mar 20;11:192. doi: 10.3389/fgene.2020.00192 (PMC7100107; doi:10.3389/fgene.2020.00192)

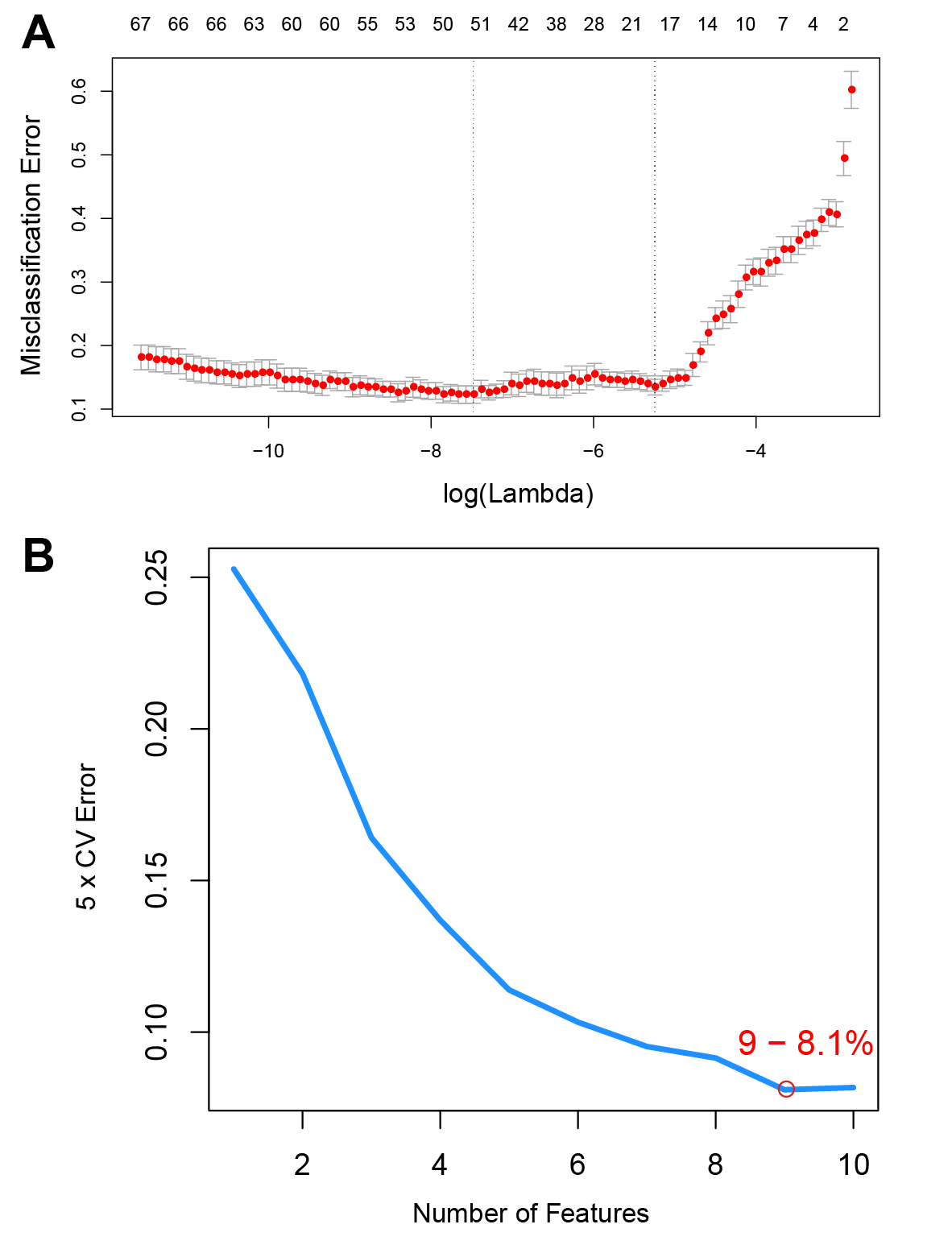

Supplement: FIGURE S1 — Cross-validation for tuning parameter selection in the LASSO logistical model (A) and SVM-RFE model (B). [file Image_1.TIF]

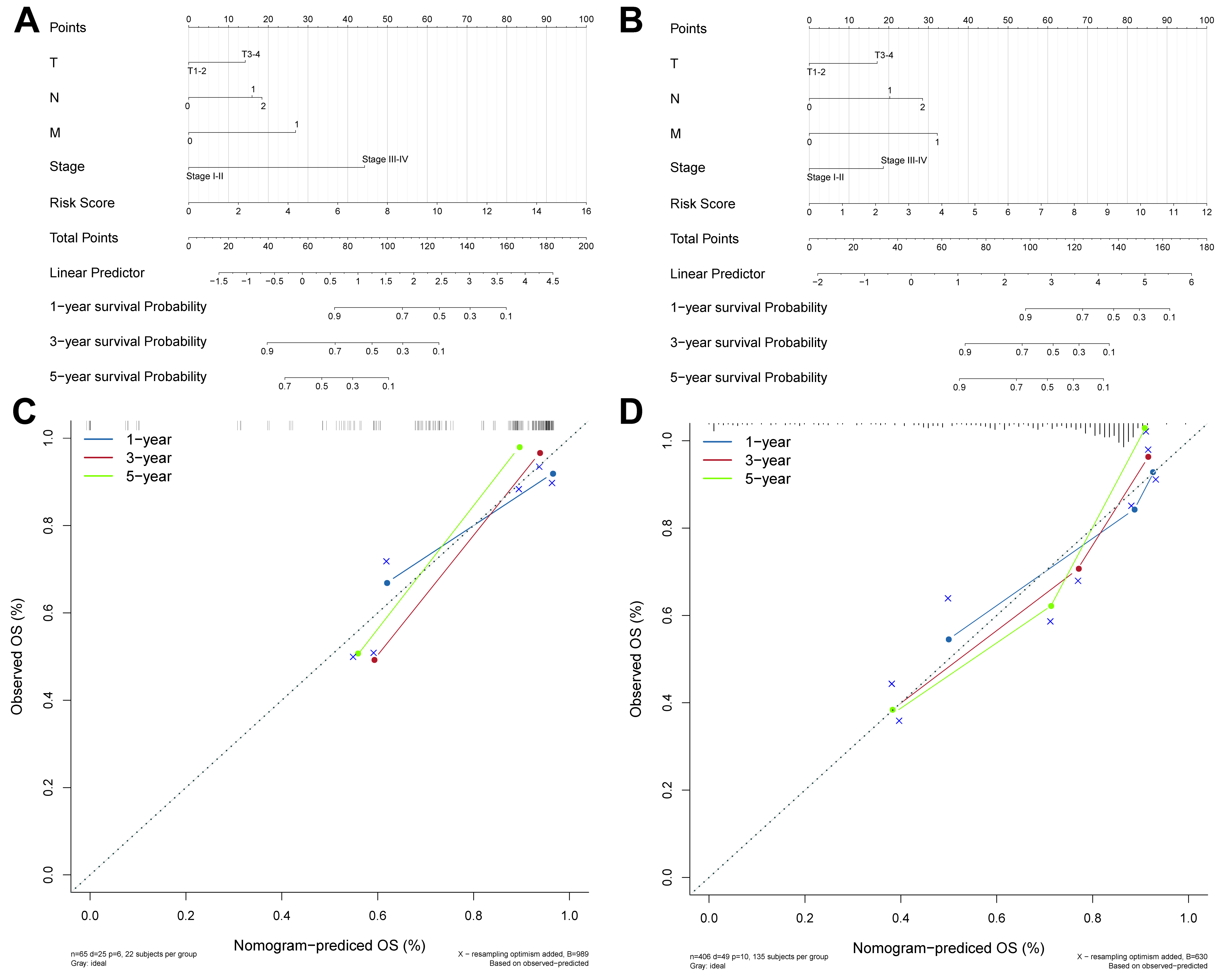

Supplement: FIGURE S2 — Establishment and validation of the composite clinicopathologic-miRNA nomogram for OS prediction. Nomograms for OS prediction in (A) testing cohort GSE29622 and (B) validation cohort TCGA-COAD. Calibration curve for the OS nomogram model in (C) testing cohort GSE29622, and (D) validation cohort TCGA-COAD. The dashed line represents the ideal nomogram, and the solid line represents the observed nomogram. [file Image_2.TIF]

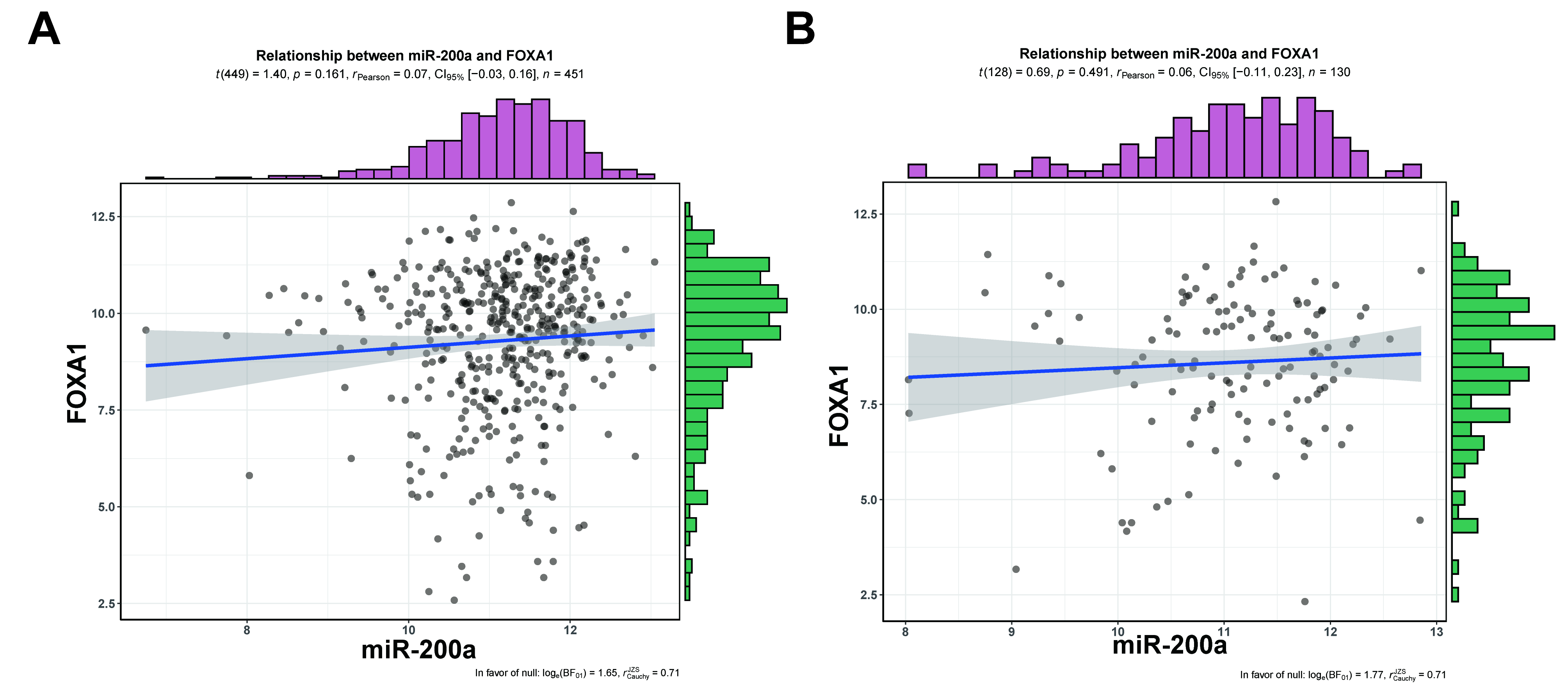

Supplement: FIGURE S3 — Scatter plots indicating the association between miR-200a and FOXA1 mRNA expression levels in (A) TCGA-COAD and (B) TCGA-Rectal cancer. [file Image_3.TIF]
