# Supplementary material for: Integrated Analysis Identifies a Nine-microRNA Signature Biomarker for Diagnosis and Prognosis in Colorectal Cancer
Source: Front Genet. 2020 Mar 20;11:192. doi: 10.3389/fgene.2020.00192 (PMC7100107; doi:10.3389/fgene.2020.00192)
Supplement: TABLE S1 — Characteristics of the public microarray datasets used in this study. [file Table_1.docx]

| **TABLE S1. Characteristics of the public microarray datasets used in this study** | | | | | | | | | | | |
| --- | --- | --- | --- | --- | --- | --- | --- | --- | --- | --- | --- |
| **Set** | **First**  **author/**  **Contributor** | **Publication year** | **Country** | **Assay type** | **Number of miRNA probes** | **Tumor site** | **Number of samples (Pairs)** | **Sample**  **Size**  **(Normal/**  **Tumor)** | **Platform** | **Source Accession** | **PMID** |
| **Training**  **set** | Li | 2015 | USA | Agilent-021827  Human miRNA  Microarray | 849 | CC | 61 pairs | 122  (61/61) | GPL10850 Agilent-021827 Human miRNA Microarray (V3) (miRBase release 12.0 miRNA ID version) | GSE48267 | 24865442 |
| **Training**  **set** | Gaedcke | 2012 | Sweden | Exiqon miRCURY  LNA Array | 894 | RC | 65 pairs | 140  (71/69) | GPL11039 Exiqon miRCURY LNA microRNA array v.9.2 Extended Version | GSE38389 | 22850566 |
| **Training**  **set** | Reid | 2012 | Italy | TaqMan MicroRNA  Array  (Applied Biosystems) | 621 | CRC | 40 pairs 37 pairs | 80  (40/40) 77  (40/37) | GPL13328 TaqMan(r) Array Human MicroRNA A Cards v2.0 GPL13329 TaqMan(r) Array Human MicroRNA B Cards v2.0 | GSE28364 | 22343615 |
| **Test set**  **for diagnosis model** | Luo | 2013 | China | Custom Microarray | 1849 | CC | 40 pairs | 80  (40/40) | GPL17496 Sun Yat-Sen University Cancer Center Human microRNA array | GSE49246 | 24239208 |
| **Validation set for diagnosis model** | Slattery | 2018 | USA | Agilent-046064 Unrestricted_  Human  _miRNA_V19.0_  Microarray (miRNA ID version) | 2030 | CRC | 752 pairs | 1513  (761/752) | GPL18402 | GSE115513 | 26740022 |
| **Test set**  **for prognosis model** | Chen | 2014 | USA | NIH Taqman Human MicroRNA Array v.2 | 664 | CC | NA | 65 | GPL11162 | GSE29622 | 22362069 |
| **Validation set for prognosis model** | TCGA | 2016 | USA | Human Illumina HiSeq 2000 | 1881 | CC | NA | 522 | Illumina HiSeq | TCGA-COAD | NA |
| Abbreviations: CC, colon cancer; CRC, colorectal cancer; RC, rectal cancer; Pairs, tumor tissues and paired adjacent noncancerous tissues from the same patient; COAD, colon adenocarcinoma. | | | | | | | | | | | |
| Comparison: colorectal cancer (CRC) vs.paired adjacent normal tissue (PANT). | | | | | | | | | | | |
