# Supplementary material for: Integrated Analysis Identifies a Nine-microRNA Signature Biomarker for Diagnosis and Prognosis in Colorectal Cancer
Source: Front Genet. 2020 Mar 20;11:192. doi: 10.3389/fgene.2020.00192 (PMC7100107; doi:10.3389/fgene.2020.00192)
Supplement: TABLE S4 — Clinical features of the CRC patients in the testing set (GSE29622), validation set (TCGA-COAD), and independent validation set. [file Table_4.docx]

Table S4. Clinical features of the CRC patients in the testing set (GSE29622), validation set (TCGA-COAD), and independent validation set.

| Characteristics | GSE29622 (n=65) | TCGA-COAD (n=522) | Independent validation cohort (n=60) |
| --- | --- | --- | --- |
| Time (months)  (mean (sd)) | 45.87 (28.58) | 47.97 (28.56) | 18.68 (10.71) |
| Age  (mean (sd)) | NA | 65.33 (10.02) | 63.8 (14.51) |
| Age (years) |  |  |  |
| < 60 | NA | 179 (31.8%) | 25 (41.67%) |
| ≥ 60 | NA | 343 (68.2%) | 35 (58.33%) |
| Sex |  |  |  |
| Female | NA | 280 (53.6%) | 21 (35%) |
| Male | NA | 242 (45.4%) | 39 (65%) |
| Local invasion |  |  |  |
| T1 | NA | 172 (33.0%) | 10 (16.7%) |
| T2 | 8 ( 12.3%) | 281 (53.8%) | 15 (25%) |
| T3 | 52 ( 80.0%) | 47 ( 9.0%) | 28 (46.67%) |
| T4 | 5 (7.7%) | 19 ( 3.6%) | 7 (11.67%) |
| Tx | NA | 3 ( 0.6%) | NA |
| Lymph node metastasis |  |  |  |
| N0 | 33 ( 50.8%) | 335 (64.3%) | 25 (41.67%) |
| N1 | 25 ( 38.5%) | 98 (18.8%) | 35 (58.33%) |
| N2 | 7 ( 10.8%) | 75 (14.4%) |  |
| N3 | NA | 2 ( 0.4%) |  |
| Nx | NA | 11 ( 2.1%) | NA |
| Distant metastasis |  |  |  |
| M0 | 47 ( 72.3%) | 353 (68.1%) | 52 (86.67%) |
| M1 | 18 ( 27.7%) | 25( 3.5%) | 8 (13.33%) |
| Mx | NA | 140 (27.0%) | NA |
| TNM stage |  |  |  |
| I | 7 ( 10.8%) | 284 (54.5%) | 9 (15%) |
| II | 22 ( 33.8%) | 126 (24.1%) | 16 (26.67%) |
| III | 18 ( 27.7%) | 85 (16.3%) | 27 (45%) |
| IV | 18 ( 27.7%) | 27 ( 5.2) | 8 (13.33%) |

NA: not available.
