# Supplementary material for: Integrated Analysis Identifies a Nine-microRNA Signature Biomarker for Diagnosis and Prognosis in Colorectal Cancer
Source: Front Genet. 2020 Mar 20;11:192. doi: 10.3389/fgene.2020.00192 (PMC7100107; doi:10.3389/fgene.2020.00192)
Supplement: DATA SHEET S1 — Supplementary experimental procedures. [file Data_Sheet_1.docx]

**Supplementary Experimental Procedures**

**Dual luciferase reporter assays**

The 3′-untranslated region (UTR) of human FOXA1 were amplified from human genomic DNA and individually inserted into the pmiR using the Xhol and Notl sites. For reporter assays, SW480 and DLD1 cells were co-transfected with wild-type (mutant) reporter plasmid and mimics (negative control) using Lipofectamine 2000 (Invitrogen). Firefly and Renilla luciferase activities were measured in cell lysates using the Dual-Luciferase Reporter Assay system. Luciferase activity was measured forty-eight hours post-transfection using dual-glo luciferase reporter system according to the manufacturer's instructions (Promega, Madison, WI, USA). Firefly luciferase units were normalized against Renilla luciferase units to control for transfection efficiency.

**Colony formation assay**

Cells were transfected with miR-200a-3p mimic or miR mimic NC, miR-200a-3p inhibitor or miR inhibitor NC, as described above. Twenty four hours later, transfected cells were trypsinized, counted, replated at a density of 800 cells/6 cm dish. Eight days later, colonies resulting from the surviving cells were fixed with 3.7% methanol, stained with 0.1% crystal violet and counted. Colonies containing at least 50 cells were scored. Each assay was performed in triplicates.

**Wound healing migration assay** ***in vitro***

SW480 and DLD1 cells were seeded in 6-well plates and incubated for twenty four hours, a linear wound was created by dragging a 200μL pipette tip through the monolayer prior to transfection. Cellular debris was removed by gentle washes with culture medium, following which transfection was performed immediately, and the cells were allowed to migrate for a further seventy two hours. The healing process was dynamically photographed after the wound was introduced using a microscope (Olympus 600 auto-biochemical analyzer, Tokyo, Japan). Migration distance was measured from images (5 fields) taken at each indicated time point. The gap size was analyzed using Image-Pro Plus 6.0 software. The residual gap between the migrating cells from the opposing wound edge was expressed as a percentage of the initial gap size.

**Transwell invasion assay**

SW480 and DLD1 cells were grown in DMEM medium containing 10% fetal bovine serum to ~50% confluence and transfected with 50 nM miR-200a-3p mimic or a NC, 100 nM miR-200a-3p inhibitor or a NC. After twenty four hours, the cells were harvested by trypsinization and washed once with Hanks’ balanced salt solution (Invitrogen). To measure cell migration, 8-mm pore size culture inserts (Transwell; Costar, High Wycombe, UK) were placed into the wells of 24-well culture plates, separating the upper and the lower chambers. In the lower chamber, 500 μL of DMEM medium containing 10% FBS was added. Then, serum-free medium containing 5 ×10^4^ cells were added to the upper chamber for Transwell assays, whereas 1×10^5^ cells were used for matrigel Transwell assays. After forty eight hours of incubation at 37°C with 5% CO_2_, the number of cells that had migrated through the pores was quantified by counting 8 independent visual fields under the microscope (Olympus, ×200 magnification), and cell morphology was observed by staining with 0.1% crystal violet. Each experiment was performed at least three times.

**Western blot analysis**

Western blot was completed as described formerly [[1](#_ENREF_1)]. Seventy-two hours after transfection, total protein was extracted from the SW480 and DLD1 cells using RIPA cell lysis reagent containing proteinase and phosphatase inhibitors (Sangon Biotech, Shanghai, China) at 4°C for 30 min. Cell lysates were centrifuged at 12,000 × g for 10 min at 4°C, and the protein concentrations of the supernatant were determined using the BCA protein assay reagent kit (Aspen). The supernatants containing total protein were then mixed with a corresponding volume of 5 × SDS loading buffer and RIPA cell lysis reagent and then heated at 100°C for 10 min. The supernatant lysates were run on 10% SDS-polyacrylamide gels (50 μg/lane), and proteins were transferred to poly (vinylidene fluoride) (PVDF) membranes (Hertfordshire, UK) by semidry electroblotting (1.5 mA/cm^2^). PVDF membranes were then incubated in blocking buffer [Tris-buffered saline (TBS) supplemented with 0.05% (vol/vol) Tween 20; TBST] containing 5% (wt/vol) skimmed milk powder for 120 min at room temperature followed by three 10 min washes in TBST. The PVDF membranes were then incubated with anti-YAP (1:1000 dilutions, Proteintech), anti-FOXA1(1:1,000 dilutions, Proteintech), , anti-E-cadherin (1:1,000 dilutions, Proteintech), anti-N-cadherin (1:1,000 dilutions, Proteintech), and anti-GAPDH (1:5,000 dilutions, Proteintech) as internal normalizers in TBST containing 5% (wt/vol) skimmed milk powder (antibody buffer) overnight at 4°C on a three-dimensional rocking table. Then the membranes were washed three times for 10 min in TBST and then incubated with goat anti-rabbit IgG conjugated to horseradish peroxidase (1:4,000 dilutions) in antibody buffer for 180 min. Finally, membranes were washed three times for 8 min in TBST and exposed to Clarity Western ECL Substrate (Bio-Rad Laboratories, Hercules, CA) for 1-2 min as described in the manufacturer's protocol , as described previously [[1](#_ENREF_1)]. The molecular weights of the bands were calculated by a comparison with prestained molecular weight markers (molecular weight range: 6,500 –250,000) that were run in parallel with the samples. Semiquantitative analysis of specific immunolabeled bands were performed using AlphaEaseFC 4.0 (Alpha Innotech)

**RRID tags for reagents.**

**Sections were stained with a rabbit polyclonal** **antibody against E-cadherin (Proteintech Group Cat# 20874-1-AP, RRID:AB_10697811)**

**Sections were stained with a rabbit polyclonal antibody against N-cadherin (Proteintech Group Cat# 13769-1-AP, RRID:AB_10598006)**

**Sections were stained with a rabbit polyclonal antibody against GAPDH (Proteintech Group Cat# 10494-1-AP, RRID:AB_2263076)**

**Sections were stained with a rabbit polyclonal antibody against YAP1 (Proteintech Group Cat# 13584-1-AP, RRID:AB_2218915)**

**Sections were stained with a rabbit polyclonal antibody against FOXA1(Proteintech Group Cat# 20411-1-AP, RRID:AB_10667003)**

**Cell lines**

**DLD-1 cell line, CLS: A colon adenocarcinoma cell line part of AstraZeneca Colorectal cell line (AZCL) panel, Cancer Cell Line Encyclopedia (CCLE) project and MD Anderson Cell Lines Project (CLS Cat# 300220/p23208_DLD-1, RRID:CVCL_0248)**

**.**

**SW480 cell line, CLS: A colon adenocarcinoma cell line part of AstraZeneca Colorectal cell line (AZCL) panel , Cancer Cell Line Encyclopedia (CCLE) project , KuDOS 95 cell line panel and MD Anderson Cell Lines Project (CLS Cat# 300302/p716_SW-480, RRID:CVCL_0546)**

**Primers used in this study**

**Table 1: Primer sequences for quantitative reverse transcription (RT)-PCR (miRNA and gene)**

| **miRNA** | **Sequence (5′ → 3′)** |
| --- | --- |
| **miR-200a-3p** | Forward: 5'-GCGCCTAACACTGTCTGGTAA -3'  Reverse: 5'-CAGCCACAAAAGAGCACAAT -3' |
| U6 | Forward:5′-CTCGCTTCGGCAGCACA -3′  Reverse:5′-TGGTGTCGTGGAGTCG -3′ |
| **Gene** | **Sequence (5′ → 3′)** |
| **Human FOXA1**  **NM_001130145.2** | Forward:5′- GCAATACTCGCCTTACGGCT-3′  Reverse:5′- TACACACCTTGGTAGTACGCC -3′ |
| **Human GAPDH**  **NM_001101** | Forward:5′- ACAGTCCATGCCATCACTG -3′  Reverse:5′- AGTAGAGGCAGGGATGATG -3′ |

1. Wang Y, Fang W, Huang Y, Hu F, Ying Q, et al. (2015) Reduction of selenium-binding protein 1 sensitizes cancer cells to selenite via elevating extracellular glutathione: a novel mechanism of cancer-specific cytotoxicity of selenite. Free Radic Biol Med 79: 186-196.
